# Orofacial pain diagnoses and their impact on Oral Health-Related Quality of life in dental patients: a cross-sectional study in Makkah, Saudi Arabia

**DOI:** 10.7717/peerj.21487

**Published:** 2026-06-26

**Authors:** Afnan A. Nassar, Abdulaziz Bakhsh, Manar Abdullah Alqahtani, Shrouq Adnan Alsharif, Ghaday Sayyam, Reyof Abdullah Alqurashi, Rawan Faleh Alqahtani, Abdelrahman M. Alhilou

**Affiliations:** 1 Division of Dental Public Health, Department of Preventative Dentistry, College of Dental Medicine, Umm Al-Qura University, Makkah, Saudi Arabia; 2 Division of Endodontics, Department of Restorative Dentistry, College of Dental Medicine, Umm Al-Qura University, Makkah, Saudi Arabia; 3 College of Dental Medicine, Umm Al-Qura University, Makkah, Saudi Arabia

**Keywords:** Orofacial pain, Endodontic pain, Quality of life

## Abstract

Orofacial pain is a prevalent clinical condition that can significantly impair patients’ quality of life (QoL). This study aimed to describe the distribution of orofacial pain diagnoses among patients attending Umm Al-Qura University Dental Hospital in Makkah and to evaluate their impact on QoL using the Oral Health Impact Profile (OHIP-14).

A cross-sectional study was conducted involving 104 adult patients presenting with orofacial pain. Diagnoses were established using standardized clinical criteria, and participants completed a structured questionnaire incorporating the OHIP-14 instrument.

Endodontic pain was the most common diagnosis (80.8%), followed by caries-related conditions (12.5%) and periodontal pain (3.8%). The median OHIP-14 score was 28, indicating a moderate negative impact on QoL. Most participants reported frequent functional limitations, physical pain, and psychological discomfort. Due to the marked imbalance in gender distribution, gender was analyzed descriptively only, and no inferential conclusions were drawn regarding sex-based differences.

Orofacial pain has a substantial impact on patients’ QoL, with endodontic conditions representing the predominant cause in this clinical setting. These findings highlight the importance of early diagnosis and targeted management to improve patient-centered outcomes. However, the clinic-based design and sample characteristics limit the generalizability of the results.

## Introduction

Orofacial pain is a common condition in dental practice that significantly affects patients’ quality of life (QoL) by disrupting their physical, psychological, social, and functional well-being (Khoo et al., 2020). It is defined as an unpleasant sensory and emotional experience associated with actual or potential tissue damage and often prompts individuals to seek professional care (Raja et al., 2020). Orofacial pain may originate from multiple anatomical structures, including teeth, oral mucosa, periodontal tissues, temporomandibular joints, muscles, sinuses, and vascular sources. Clinically, it can manifest as acute or chronic pain (Scrivani & Spierings, 2016). Acute dental pain typically serves as a protective warning signal and resolves following appropriate treatment, whereas chronic pain persists for more than three months and often requires prolonged management (Renton, 2018).

Acute intraoral pain is the most common presentation and is frequently associated with conditions such as dental pulpitis, pericoronitis, trauma, and infection. Reported prevalence is high, with pulpitis reaching up to 91% in some populations (Kiranmayi, Anumala & Kirubakaran, 2019) and pericoronitis up to 70% (Rezvi, Balasubramaniam & Chaudhary, 2020). Given its impact on daily functioning, social participation, and work productivity, effective management of orofacial pain through accurate diagnosis and timely intervention is essential for improving QoL and overall well-being (Dagher et al., 2019; AlSahman et al., 2025; Agnese et al., 2025).

Evidence from North America indicates that orofacial pain, particularly acute dental pain and temporomandibular disorders (TMDs), has a significant negative impact on oral health-related QoL. Elevated Oral Health Impact Profile (OHIP) scores consistently demonstrate impaired daily functioning and well-being among affected individuals (Moufti et al., 2011; Rener-Sitar et al., 2013; Petričević et al., 2009). These findings underscore the considerable burden that orofacial pain imposes. Despite increasing global awareness, there remains limited evidence on the prevalence and impact of orofacial pain on QoL in Saudi Arabia, particularly in Makkah City.

Orofacial pain extends beyond a clinical symptom and can significantly impair daily functioning and overall well-being. Patients frequently report difficulty eating, speaking, and sleeping as well as increased psychological distress and reduced social participation. These effects have been consistently associated with reduced oral health-related QoL (OHRQoL) (Moufti et al., 2011; Slade, 1997; Patel et al., 2025).

Patient-reported outcome measures such as the Oral Health Impact Profile (OHIP-14) are therefore essential, as they capture subjective experiences of pain and its functional and psychosocial consequences-dimensions that may not be fully reflected in clinical assessment alone (Slade, 1997). Evaluating OHRQoL provides a more comprehensive understanding of disease burden and supports patient-centered care.

Contextual and cultural factors may further influence pain perception and reporting. In Saudi Arabia, differences in healthcare access, socioeconomic status, and health-seeking behaviors—including delayed care and reliance on over-the-counter medications—may shape how patients experience and manage orofacial pain. Previous studies have highlighted the role of those factors in influencing oral health outcomes and pain-related behaviors (Alshammari et al., 2021; Renger, Patzke & Alrayes, 2025).

This study therefore aimed to investigate the distribution of orofacial pain diagnoses among patients attending Umm Al-Qura University Dental Hospital in Makkah City and to assess their impact on OHRQoL using OHIP-14.

## Materials & Methods

This clinic-based cross-sectional observational study involved patients presenting with pain at the dental care and screening clinic, Faculty of Dental Medicine, Umm Al-Qura University, Makkah, Kingdom of Saudi Arabia, between October 2023 and January 2024. Inclusion criteria were adult patients aged 18 years or older presenting with intraoral or extraoral pain who provided written informed consent. The clinic examines approximately 40 patients per month. Of these, 104 participants met the inclusion criteria and were enrolled, representing approximately 65% of the screened population. Patients were excluded if they were unable to adequately understand or respond to the questionnaire due to language barriers (*e.g.*, non-Arabic or non-English speakers), cognitive impairment, or other communication limitations. The study was conducted in accordance with the Declaration of Helsinki, and ethical approval was obtained from the Institutional Review Board of Umm Al-Qura University (Approval No. HAPO-02-K-012-2023-09-1769).

Clinical examinations were performed by calibrated sixth-year dental students under the direct supervision of consultants. Calibration and training were completed prior to data collection. Each assessment included a comprehensive dental and medical history. The diagnostic process involved patient questioning followed by confirmatory clinical tests administered.

Dental diagnoses were recorded using dental charts and the decayed, missing, and filled teeth (DMFT) index, a validated measure of oral health status (Moradi et al., 2019). Periodontal status was assessed using the 2017 New Periodontal Classification system (Caton et al., 2018). Endodontic diagnoses followed the American Association of Endodontists (AAE) diagnostic guidelines  (Glickman, 2009). Temporomandibular disorders (TMDs) were diagnosed using the Diagnostic Criteria for Temporomandibular Disorders (DC/TMD), encompassing arthralgia, myalgia, local myalgia, myofascial pain (with or without referral), disc displacement disorders, degenerative joint disease, subluxation, and TMD-related headaches (Schiffman et al., 2014).

Participants completed a three-part questionnaire comprising demographic information, pain characteristics, and the OHIP-14 (Slade, 1997). Pain characteristics were assessed using a structured questions adapted from Cohen’s Pathways of the Pulp (10th edition), including pain onset, duration, intensity, localization, triggering factors (*e.g.*, thermal stimuli, mastication), and relieving factors.

The questionnaire was developed in English and translated into Arabic using a forward–backward translation process by bilingual experts. A pilot study was conducted to ensure clarity and cultural appropriateness. Content validity was evaluated by a panel consisting of one associate professor in dental public health and four practicing dentists to ensure adequate coverage of the study constructs. Face validity was established through expert review to assess clarity, language, grammar, logical consistency, and overall structure. Reliability testing yielded a Cronbach’s alpha coefficient of 0.80, indicating good internal consistency.

Data were entered and analyzed using IBM SPSS Statistics version 23.0. Categorical variables were analyzed using the Chi-square test or Fisher’s exact test, as appropriate. Continuous variables were assessed for normality and compared using the Kruskal–Wallis test. Associations between demographic variables and pain diagnoses were evaluated, with *p*-values were reported. A *p*-value of <0.05 was considered statistically significant.

Responses to each OHIP-14 item were scored on a scale from 0 to 5. Total OHIP-14 scores were calculated by summing all 14 items, yielding a possible range of 0 to 56. Higher total scores indicated worse OHRQoL, whereas lower scores indicated better OHRQoL (Slade, 1997). Because OHIP-14 scores were not normally distributed, a non-parametric Kruskal–Wallis test was used to compare scores across diagnostic groups. Statistical significance was set at *p* < 0.05.

## Results

A total of 104 patients participated, with females comprising 90.4% (*n* = 94) of the sample. Most participants were non-Saudi (79%) and reported an income below 5,000 Saudi Riyals (96.2%). Demographic characteristics are presented in Table 1.

**Table 1:** Demographic characteristics.

	Category	Frequency (*n*)	Percentage (%)
Gender	Male	10	9.6
	Female	94	90.4
Nationality	Saudi	21	20.2
	Non-Saudi	83	79.8
Income	<5,000 SAR	100	96.2
	5,000–10,000 SAR	4	3.8
	>10,000 SAR	0	0

Endodontic-related pain was the most prevalent diagnosis (80.8%), most commonly triggered by cold stimuli (59.6%). Caries-related pain accounted for 12.5% of cases, periodontal pain 3.8%, TMD 1.9%, and oral pathology (ulcers) 1%. Detailed findings from the diagnostic assessment and pain characteristics are presented in Table 2.

**Table 2:** Pain characteristics and origin.

	Variable	Frequency (*n*)	Percentage (%)
Can you localize the pain	Yes	99	95.2
	No	5	4.8
The pain initiated by	Cold	62	59.6
	Heat	4	3.8
	Sweet	4	3.8
	Spontaneous pain	2	1.9
	Mastication	28	26.9
	Other/unspecified trigger	1	0.9
	Stress	3	2.7
Relieved by	Cold	4	3.7
	Heat	0	0.0
	Over the counter medication	60	57.7
	Narcotic medication	3	2.9
	Herbal/traditional medicine	2	1.9
	None	35	33.7
Pain location	Anterior teeth	34	32.7
	Posterior teeth	65	62.5
	Joint & muscle	2	1.9
	Periodontium	3	2.9
Pain origin based on diagnostic tests	Endodontic	84	80.8
	Periodontal	4	3.8
	Caries	13	12.5
	TMD	2	1.9
	Ulcer	1	.96

Chi-square analysis revealed no consistent statistically significant associations between demographic variables and orofacial pain characteristics or diagnoses (*p* > 0.05). Although some associations reached statistical significance—specifically gender and diagnosis (*p* = 0.023) and nationality and type of diagnostic test (*p* = 0.038)—these findings were interpreted cautiously due to the imbalance in sample distribution. Detailed diagnostic distribution are presented in Table 3.

**Table 3:** Orofacial pain-related diagnosis.

	Variable	Frequency (*n*)	Percentage (%)^a^
Pulpal	Reversible pulpitis	5	5.5
	Symptomatic irreversible pulpitis	39	43.3
	Asymptomatic irreversible pulpitis	30	36
	Pulp necrosis	7	7.9
	Previously treated	3	3.4
	Previously initiated therapy	1	1
Periapical	Symptomatic apical periodontitis	60	71.4
	Asymptomatic apical periodontitis	12	14.3
	Acute apical abscesses	0	0
	Chronic apical abscesses	4	4.8
	Normal apical tissue	8	9.5
Periodontal	Periodontal health, gingival diseases, and condition	0	0
	Periodontitis	1	25
	Other conditions affecting the periodontium	3	75
	Peri implant diseases and condition	0	0
Carious lesion	Class I	0	0
	Class II	6	42.9
	Class III	3	21.4
	Class IV	3	21.4
	Class V	2	14.3
TMD	Arthralgia	1	50
	Disc displacement	0	0
	Myofascial pain	1	50

**Notes.**

a Percentages are calculated within each diagnostic category.

Comprehensive QoL findings for all participants are summarized in Table 4. Responses to individual OHIP-14 items indicated substantial functional, physical, and psychosocial impacts, with most participants reporting “very often” or “fairly often” across multiple domains. More than 80% reported oral physical pain, 70% psychological discomfort, 85% physical limitation, 80% psychological impact, 70% social discomfort, and 80% reduced life satisfaction.

**Table 4:** Self-reported quality of life and oral health.

Item	Very often	Fairly often	Occasionally	Never	Hardly ever
Trouble pronouncing words	17 (16.3%)	34 (32.7%)	0	38 (36.5%)	15 (14.4%)
Worsened taste	20 (19.2%)	37 (35.6%)	0	39 (37.5%)	8 (7.7%)
Painful aching	44 (42.3%)	47 (45.2%)	0	9 (8.7%)	3 (2.9%)
Uncomfortable eating	56 (53.8%)	37 (35.6%)	0	8 (7.7%)	4 (3.8%)
Self-conscious	35 (33.7%)	43 (41.3%)	0	25 (24.0%)	2 (1.9%)
Felt tense	29 (27.9%)	48 (46.2%)	0	18 (17.3%)	9 (8.7%)
Unsatisfactory day	43 (41.3%)	47 (45.2%)	0	10 (9.6%)	4 (3.8%)
Interrupted meals	44 (42.3%)	47 (45.2%)	0	10 (9.6%)	2 (1.9%)
Difficulty relaxing	41 (39.4%)	47 (45.2%)	0	13 (12.5%)	3 (2.9%)
Embarrassed	33 (31.7%)	48 (46.2%)	0	20 (19.2%)	3 (2.9%)
Irritable	29 (27.9%)	38 (36.5%)	0	32 (30.8%)	5 (4.8%)
Difficulty working	34 (32.7%)	52 (50.0%)	0	17 (16.3%)	1 (1.0%)
Life less satisfying	25 (24.0%)	55 (52.9%)	0	23 (22.1%)	2 (1.9%)
Unable to function	33 (31.7%)	55 (52.9%)	0	13 (12.5%)	3 (2.9%)

**Figure 1:**
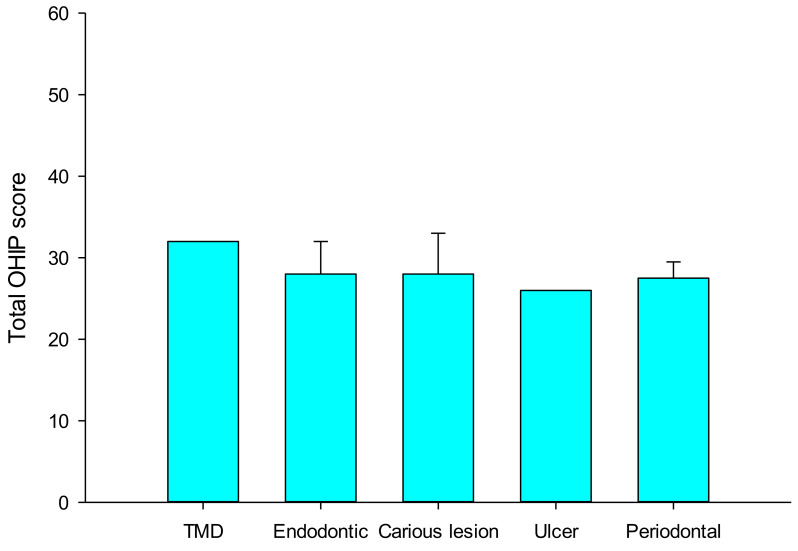
Bar graph showing the distribution of Oral Health Impact Profile (OHIP)-14 scores across the various orofacial pain diagnoses.

Orofacial pain had a measurable impact on patients’ QoL. When OHIP-14 scores were analyzed by diagnostic category, patients with TMD reported the highest median score (32.0; IQR: 0), whereas those with periodontal-related pain reported the lowest median score (27.5; IQR: 5.0). The distribution of OHIP-14 scores across diagnostic categories is shown in Fig. 1. However, the differences between groups were not statistically significant (*p* = 0.896).

A statistically significant association was observed between gender and endodontic pain, with female patients being more likely to report endodontic pain (79.80%; *p* = 0.023). Additionally, a significant association was found between non-Saudi nationality and the use of over-the-counter medications (*p* = 0.05).

## Discussion

This cross-sectional study evaluated the distribution and impact of orofacial pain among dental patients in Makkah. The primary finding was that endodontic (odontogenic) pain accounted for 80.8% of cases, substantially exceeding other sources such as caries-related pain (12.5%), periodontal pain (3.6%), and TMD-related pain (1.9%). This pattern is consistent with global evidence indicating that pulpitis and endodontic infections account for approximately 60%–82% of dental pain emergencies (Estrela et al., 2011; Rechenberg et al., 2016; Alhilou et al., 2023).

Pulpal pain is primarily caused by dental caries, which remains the leading cause of dental pain worldwide (Delgado-Pérez et al., 2024). In Saudi Arabia, where dental caries affects more than 90% of the population, patients commonly seek care for toothache related to decay and pulpal inflammation (Alshammari et al., 2021). In the present study, 59.5% of involved pain triggered by cold stimuli, consistent with clinical symptoms of reversible and irreversible pulpitis. These findings underscore the role of untreated dental caries and its complications as major contributors to orofacial pain.

In our study cohort, TMD-related pain accounted for 1.9%, which is lower than comparable studies reporting a prevalence of 7–10% in the general population (Hadlaq et al., 2019). This low representation likely reflects the dental hospital’s focus on acute conditions, whereas patients with chronic TMD may seek care in other settings. Nonetheless, TMD-related pain in our study was associated with substantial impact on QoL. The patient with TMD had the highest median OHIP-14 score (32); however, patients with endodontic or other types of pain reported similarly high scores, indicating poor OHRQoL across conditions. This difference was not statistically significant, likely due to the small sample size.

Our findings are consistent with previous research in a Saudi population showing that patients with TMD experienced significantly lower QoL across all OHIP-14 domains compared to controls (AlSahman et al., 2025). The lack of statistically significant differences in OHIP-14 scores across diagnostic categories suggests that both odontogenic and non-odontogenic pain can substantially affect QoL. It further indicates that once pain severity reaches a certain threshold, its impact on QoL may be consistently high regardless of etiology. For example, acute endodontic pain can be severe and disruptive, affecting work, sleep, and mood in ways comparable to chronic TMD pain, despite its shorter duration.

The OHIP-14 results in this study further highlight the considerable impact of orofacial pain on daily functioning. Participants reported a median score of 28 (out of 56), indicating a moderate level of impairment in functional and psychosocial well-being. Higher OHIP-14 scores reflect frequent experiences of pain, difficulty eating, disrupted sleep, and increased self-consciousness. A median score of 28 is notably higher than population norms, suggesting that even acute episodes of dental pain can substantially impair QOL. This finding aligns with international studies linking dental pain to reduced OHRQoL  (Agnese et al., 2025; Patel et al., 2025; Farid et al., 2025).

Our findings also suggest observable demographic patterns in pain experience and coping behaviors. A higher proportion of female participants reported endodontic pain, consistent with previous literature indicating greater pain reporting and healthcare-seeking behavior among women (Delgado-Pérez et al., 2024). However, this observation should be interpreted cautiously due to the predominance of female participants in the sample. This imbalance may reflect differences in healthcare utilization, with women potentially more likely to seek dental care or participate in clinical research, but it may also introduce selection bias and limit generalizability. Accordingly, gender-related findings should be considered exploratory.

Other studies of chronic orofacial pain, including TMD, also report a female predominance (LeResche, 1997; Schmid-Schwap et al., 2013).Biological and psychosocial factors, such as pain perception, hormonal influences and healthcare-seeking behaviors, may explain this disparity (Delgado-Pérez et al., 2024; Alhilou et al., 2021). In the present study, the higher prevalence of endodontic pain among women may reflect regional patterns of healthcare utilization, However, no significant difference in OHIP-14 scores were observed between genders, suggesting that the impact of pain on QoL is comparable.

Another notable finding was the association between patient nationality and pain management behavior. Non-Saudi participants were significantly more likely to use over-the-counter medications prior to seeking professional care. This pattern likely reflects disparities in access to healthcare. Saudi citizens generally have greater access to subsidized dental services, whereas non-Saudis may face financial or logistical barriers, leading delayed care and increased reliance on self-medication. This interpretation is supported by existing literature demonstrating that lower-income and underserved populations are more likely to delay treatment and experience higher burdens of dental pain (Renger, Patzke & Alrayes, 2025; Wu et al., 2025).

This study provides clinically relevant insights into the distribution and impact of orofacial pain in a teaching hospital setting, contributing to a relatively underexplored area in dental public health. The use of a structured questionnaire incorporating the OHIP-14, along with acceptable internal consistency (Cronbach’s alpha = 0.80), supports the reliability of the findings. Furthermore, data collection in a real-world clinical setting and the integration of patient-reported outcomes with clinical assessment enhance the practical applicability and ecological validity of the results.

However, several limitations that should be considered when interpreting the findings. First, the clinic-based cross-sectional design and use of consecutive sampling may limit the generalizability. Second, the predominance of female and non-Saudi participants may introduce selection bias and affect representativeness. Third, although the questionnaire demonstrated acceptable reliability, a validated Arabic version of the OHIP-14 was not used, which may limit comparability with other studies. Additionally, the relatively small sample size and the presence of very small subgroups (*e.g.*, TMD and oral pathology), reduces statistical power and limit meaningful comparisons across diagnostic categories. Consequently, differences in QoL across groups should be interpreted with caution. Future research with larger, more diverse, and more representative samples is needed to improve the external validity and enable more robust subgroup analyses.

## Conclusions

The high prevalence of endodontic-related pain observed in this study is consistent with global evidence identifying dental caries and pulpitis as major contributors to orofacial pain. Demographic factors such as gender and nationality may influence pain experience and management, highlighting the importance of considering broader social determinants in oral healthcare delivery. Regardless of etiology, orofacial pain was found to have a substantial negative impact on OHRQoL, underscoring that dental pain extends beyond a localized clinical condition to affect functional and psychological well-being. Addressing both the clinical and social dimensions of orofacial pain is essential for improving patient-centered care outcomes.

##  Supplemental Information

10.7717/peerj.21487/supp-1 Supplemental Information 1 Survey

10.7717/peerj.21487/supp-2 Supplemental Information 2 STROBE Cheklist

10.7717/peerj.21487/supp-3 Supplemental Information 3 Raw Data

10.7717/peerj.21487/supp-4 Supplemental Information 4 Codebook

## Additional Information and Declarations

### Data Availability

The following information was supplied regarding data availability: The Raw Data is available in the Supplemental Files.
