# Supplementary material for: Orofacial pain diagnoses and their impact on Oral Health-Related Quality of life in dental patients: a cross-sectional study in Makkah, Saudi Arabia
Source: PeerJ. 2026 Jun 26;14:e21487. doi: 10.7717/peerj.21487 (PMC13312972; doi:10.7717/peerj.21487)
Supplement: Supplemental Information 1 [file peerj-14-21487-s001.docx]

# Oral Pain and Quality of Life Survey (patient survey)

We are sixth-year dental students at the Faculty of Dentistry, Umm Al-Qura University. This questionnaire aims to assess oral and facial pain and its impact on quality of life among residents of the Makkah city of Saudi Arabia, including both citizens and non-citizens.

Participation is voluntary. Completing this questionnaire indicates your consent to participate. All information is confidential and used for research purposes only.

Informed Consent
By completing this questionnaire, you confirm that you have read the information above and agree to participate in this study.

Section A: Demographic Information
1. Name:
2. Age:
3. Gender: Female / Male
4. Nationality: Saudi / Non-Saudi
5. Monthly Income: <5000 / 5000–10000 / >10000 SAR
6. Marital Status: Married / Widowed / Divorced / Single
7. Educational Level: Intermediate or less / High school / Bachelor / Postgraduate

Section B: Pain Characteristics
1. Type of pain experienced: Tooth / Gum / Chewing / Facial / TMJ
2. Pain onset: <3 months / ≥3 months
3. Pain description: Mild / Sharp / Throbbing / Continuous / Burning / Stabbing / Electric
4. Can you identify pain location? Yes / No
5. Pain triggers: Cold / Heat / Sugar / Touch / Chewing / Position / Stress
6. Pain relief: Cold / Heat / OTC drugs / Prescription / Herbal

Section C: Quality of Life
Responses: Very often / Sometimes / Hardly ever / Never / Do not know
1. Difficulty pronouncing words?
2. Worsened sense of taste?
3. Pain in mouth?
4. Discomfort eating?
5. Feeling embarrassed?
6. Feeling tense?
7. Unsatisfactory day?
8. Interrupted meals?
9. Difficulty relaxing?
10. Slight embarrassment?
11. Irritability?
12. Difficulty usual activities?
13. Life less satisfying?
14. Unable to perform daily activities?
