# Supplementary material for: Orofacial pain diagnoses and their impact on Oral Health-Related Quality of life in dental patients: a cross-sectional study in Makkah, Saudi Arabia
Source: PeerJ. 2026 Jun 26;14:e21487. doi: 10.7717/peerj.21487 (PMC13312972; doi:10.7717/peerj.21487)
Supplement: Supplemental Information 4 [file peerj-14-21487-s004.docx]

**Coding form:**

Gender:

Male=1

Female= 2

Nationality:

Saudi=1

Non=2

Income:

Less than 5K =1

5k – 10k =2

More=3

Q1: Localize pain=

Yes=1

No=2

Q2: Initiated by:

Cold=1

Hot=2

Sugar=3

Touch=4

Mastication=5

Laying down=6

Depression or stress=7

Q3: Relive with:

Cold=1

Hot: 2

OPC=3

NCM=4

Herbal=5

Non=6

Q4: Examiner:

Dr.Afnan: 1

Steudent:2

Q5: Tooth number:

Ant.:1

Post:2

Joint & Muscles :3

Periodontium: 4

Q6: Type of test:

Endo ice:1

Perio probe:2

WHO Criteria: 3

DCTMD:4

Other= 5

Q7: Diagnosis:

Endo:1

Perio:2

Resto:3

Orofacial pain:4

Oral pain(ulcer):5

Other:6

Q8: Questionnaire done :

Before:1

After: 2

Q9: Have you had trouble pronouncing any words because of the problem with your teeth or mouth?

Very often = 1

Fairly often=2

Never=3

Don’t know=4

Q10: Have you ever felt that your sense of taste has worsened because of a problem with your teeth or mouth?

Very often = 1

Fairly often=2

Never=3

Don’t know=4

Q11: have you had been aching in your mouth?

Very often = 1

Fairly often=2

Never=3

Don’t know=4

Q12: Have you found it uncomfortable to eat any food because of the problem with your teeth or mouth?

Very often = 1

Fairly often=2

Never=3

Don’t know=4

Q13: Have you been self-conscious because of your teeth or mouth?

Very often = 1

Fairly often=2

Never=3

Don’t know=4

Q14: Have you felt tense because of a problem with your teeth or mouth?

Very often = 1

Fairly often=2

Never=3

Don’t know=4

Q15: Has your day been unsatisfactory because of a problem with your teeth or mouth?

Very often = 1

Fairly often=2

Never=3

Don’t know=4

Q16: Have you had to interrupt meals because of problems with your teeth or mouth?

Very often = 1

Fairly often=2

Never=3

Don’t know=4

Q17: Have you found it is difficult to relax because of a problem with your teeth or mouth?

Very often = 1

Fairly often=2

Never=3

Don’t know=4

Q18: Have you been a bit embarrassed because of a problem in your teeth or mouth?

Very often = 1

Fairly often=2

Never=3

Don’t know=4

Q19: Have you been a bit irritable with other people because of a problem with your teeth or mouth?

Very often = 1

Fairly often=2

Never=3

Don’t know=4

Q20: Have you had difficulty doing your usual job because of a problem in your teeth or mouth?

Very often = 1

Fairly often=2

Never=3

Don’t know=4

Q21: Have you felt that life in general was less satisfying because of a problem with your teeth or mouth?

Very often = 1

Fairly often=2

Never=3

Don’t know=4

Q22: Have you been totally an able to function because of a problem in your teeth or mouth?

Very often = 1

Fairly often=2

Never=3

Don’t know=4

Endodontic diagnosis.

Periapical diagnosis.

Reversible pulpitis = 1

Symptomatic irreversible pulpitis = 2 Symptomatic apical periodontitis= 1

Asymptomatic irreversible pulpitis = 3 Asymptomatic apical periodontitis= 2

Pulp necrosis = 4 Acute apical abscesses = 3

Previously treated = 5 Chronic apical abscesses=4

Previously initiated therapy =6. None of the above=7

None of the above =7

Optative diagnosis:

Class l =1

Class ll =2

Class lll=3

Class lV=4

Class V = 5

None of above =7

Perio diagnosis:

Periodontal health, gingival diseases, and condition= 1

Periodontitis =2

Other conditions affecting the periodontium =3

Peri implant diseases and condition=4

None of above = 7

TMD diagnosis :

Arthralgia = 1

Disc displacement. =2

Myofascial Paine =3

Non of the above =7
